# Combined Minimally Invasive Treatment of Teeth Discoloration Caused by Occupational Exposure to Bronze Alloy

**DOI:** 10.1155/2022/3399120

**Published:** 2022-11-23

**Authors:** Shahram Amirifar, Elham Ahmadi, Elmira Najafrad, Niyousha Rafeie

**Affiliations:** ^1^Department of Restorative Dentistry, School of Dentistry, Tehran University of Medical Sciences, Tehran, Iran; ^2^Dental Research Center, Dentistry Research Institute, Restorative Dentistry, School of Dentistry, Tehran University of Medical Sciences, Tehran, Iran; ^3^Dental Research Center, Dentistry Research Institute, School of Dentistry, Tehran University of Medical Sciences, Tehran, Iran

## Abstract

Recently, dental bleaching has been frequently sought by patients to improve the appearance and color of the teeth. Among various treatment options, in-office bleaching is commonly preferred by patients with severely discolored teeth due to its fast aesthetic results. In addition, other pretreatment methods, such as air-powder polishing, have been reported to increase the efficacy of bleaching treatment. Compared to other causes of tooth discoloration, occupational tooth discoloration caused by bronze alloy and further treatment have not been sufficiently documented in the literature. In the present case report, we explain a case of tooth discoloration following exposure to a bronze alloy and a conservative clinical approach used for the management of tooth discoloration. A 15-year-old male patient who worked in a foundry was presented with tooth discoloration. At the first session, a rubber-cap prophylaxis was performed. After one week, air-powder polishing was used to remove the remaining stains. Finally, at the third session, two cycles of bleaching were performed using hydrogen peroxide gel 35%, each for 20 minutes. Photographs were taken at the end of each session and used for visual evaluation. The final result was aesthetically satisfactory.

## 1. Introduction

In recent years, the appearance and color of dentition have become a significant concern among patients. As a result, the number of patients seeking treatment for tooth discoloration has increased. Many factors have been attributed to tooth discoloration, including genetic disorders such as amelogenesis imperfecta, dentinogenesis imperfecta, congenital erythropoietic, and porphyria alkaptonuria. In addition, high doses of fluoride, tetracycline use, trauma, pulp hemorrhage, and occupational exposure to specific metals (such as iron and copper) are other contributing factors to tooth discoloration. Occupational exposure to metallic or drug consumption containing metallic salts is one of the major causes of external tooth staining [1], and the black staining in people consuming iron supplements and foundry workers has been well documented in the literature [2, 3]. It has been reported that copper can also cause green staining in factory workers and patients using mouth rinses containing copper [4–6]. Tooth staining caused by potassium permanganate, silver nitrate salt, and stannous fluoride has also been reported.

Recently, many tooth whitening methods have been introduced to dental practice; whitening toothpastes, gels and strips, whitening mouth rinses, as well as bleaching, are some of these methods [7]. Most of them contain hydrogen peroxide with different concentrations to exert their whitening effect [8]. Among these methods, in-office bleaching is commonly chosen by patients with severe discoloration due to its fast and immediate results [8]. It is believed that the application of an air-powder polishing (APP) device with sodium bicarbonate before in-office bleaching might increase the efficacy of the bleaching treatment. Moreover, air polishing requires less time compared to traditional polishing methods. While polishing with a rubber cup and prophylaxis paste increases the enamel roughness and removes the fluoride-rich enamel, air polishing is safe on the tooth structure with less abrasivity compared to the traditional polishing method and does not cause significant enamel loss [9].

Due to the limited documentation of tooth discoloration caused by bronze, subsequent treatment outcome, and follow-up results, the present clinical case report aimed to record in detail the treatment procedure and follow-up results of a tooth discoloration caused by bronze alloy in a foundry worker.

## 2. Case Presentation

**Figure 1:**
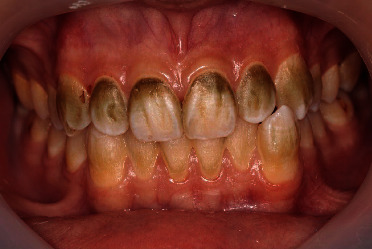
Tooth discoloration caused by occupational exposure to copper in a 15-year-old male patient.

A 15-year-old male patient presented with moderate to severe green stains on the upper and lower anterior teeth (Figure 1). The patient worked 40 hours per week for 24 months. After 5 months, he noticed the green stain deposits on his anterior teeth. On the first appointment, he expressed the desire for whiter teeth as well as maintaining tooth structure as much as possible. The medical history was unremarkable; he was not taking any medications and had no significant family history.

**Figure 2:**
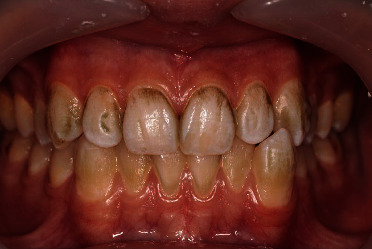
Teeth after prophylaxis with pumice powder using a low-speed handpiece at the end of the first appointment.

The treatment plan and the reason for the discoloration were explained to the patient, and informed consent was secured. Evaluation of panoramic radiography revealed no root canal or restorative treatment in the anterior teeth. At the first appointment, after taking the patient's guardian's informed consent, the baseline color of the teeth was recorded by photography, and teeth prophylaxis was performed with pumice powder using a low-speed headpiece at a pace of 2500–3000 rpm and each tooth surface was polished for 5 s. Another photograph was taken immediately after the prophylaxis procedure (Figure 2). Stains were mostly removed; however, discoloration was still present in deep fissures and the cervical region. At the end of the session, modified bass brushing technique and flossing technique were demonstrated to the patient. The patient was advised to brush 2 times a day, each time for at least 20 minutes and floss daily.

**Figure 3:**
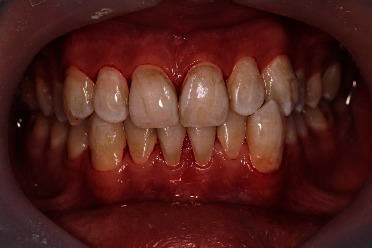
Teeth after air polishing treatment using air flow handy device with sodium bicarbonate.

One week later, another appointment was scheduled. In the second appointment, the air polishing treatment was performed using the Airflow Handy device (EMS, Nyon, Switzerlan). Sodium bicarbonate was used as an air-powder polishing powder (AIRFLOW® Plus, EMS Electro Medical Systems, Nyon, Switzerland). The device nozzle was held 3 mm from the tooth surface. The spray was applied for 5 s on each tooth surface with a constant circular movement for each surface [9]. An image was taken after the treatment for further evaluation (Figure 3). Visual evaluation revealed that stains were effectively removed. However, some degree of discoloration was still present. Another appointment was scheduled in 1 week.

**Figure 4:**
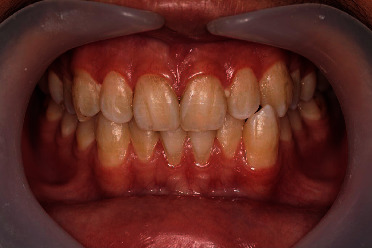
Teeth after bleaching with hydrogen peroxide whitening gel at the end of the final appointment.

**Figure 5:**
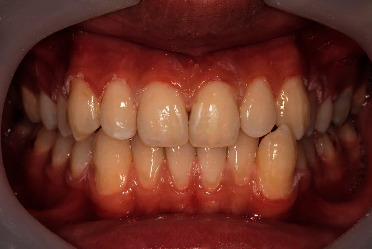
Teeth after the treatment at the follow-up session 6 months after the initial treatment.

In the third appointment, hydrogen peroxide whitening gel at 35% concentration (Whiteness HP, FGM Co., Connecticut, USA) was prepared by mixing phases 1 and 2 in a ratio of 3 : 1 according to the manufacturer's instructions. A gingival barrier was used to protect the gingiva and adjacent tissues. A desensitizing agent (Desensibilize KF 2%) was applied on the tooth surfaces and was removed after 10 min. The bleaching gel was then applied in 2 mm thickness on the buccal surfaces of the anterior teeth in both arches. The gel remained on surfaces for 20 min and then was suctioned by using fine suction cannula. The tooth surfaces were thoroughly washed. This procedure was repeated for a second time in the same appointment (Figure 4). The patient was advised to avoid foods and beverages that are capable of staining the teeth, such as tea, coffee, and blueberries. The patient and his guardian were satisfied with the final results. At the end, the patient's guardian was asked if he would consent to use his son's information in a journal article, and an informed consent form was signed by him. The patient was asked to come 6 months after the treatment for a follow-up evaluation.  Figure 5 shows the treatment result in the follow-up session after 6 months.

It should be noted that the patient changed his job after the first session of the treatment and there was no risk of continuous occupational exposure to bronze during the course of treatment.

## 3. Discussion

The present case report utilized rubber cap prophylaxis and air-powder polishing in combination with conventional in-office bleaching treatment to improve the aesthetic results of whitening treatment in a patient suffering from tooth discoloration caused by bronze alloy.

Metallic salts are considered one of the main contributing factors in cases of external tooth discoloration. A few tooth discoloration cases caused by iron and copper have been documented in the literature. Donoghue and Ferguson reported a case of tooth discoloration caused by superficial copper staining in a brass foundry worker [6].

Two main mechanisms have been proposed to explain tooth discoloration caused by metallic compounds. According to the first mechanism, tooth discoloration is caused by the production of sulfide salts of the metallic salts on tooth surfaces. However, there is little evidence to support this theory [10]. According to the second theory, tooth discoloration results from N1-type of discoloration. It is believed that when the enamel is in contact with saliva, the negative charge of the enamel is neutralized by ions present in the saliva and a layer known as the stern layer is formed on the enamel surface. In the presence of metallic ions such as copper, iron, and nickel in the saliva, these ions accumulate on the enamel and may form dental stains on the tooth surfaces [11].

We believe that the patient's exposure to bronze fume and dust during working hours has caused observable tooth discoloration. Since bronze alloy is roughly composed of 88% copper, it is probable that copper particles present in bronze fumes have adhered to the tooth surface and caused green discoloration. Insufficient respiratory protection or the use of low-quality masks might be the reason for increased exposure of the patient to these particles and subsequent tooth discoloration. It should be noted that in addition to tooth discoloration, inhalation of copper particles can cause more severe side effects such as respiratory tract and sinus irritation, palpebral edema, conjunctivitis, and cough [12].

Among various proposed tooth whitening methods, the office bleaching method has many advantages, including the prevention of soft tissue irritation and immediate and fast aesthetic outcomes [13]. It is believed that the bleaching agents penetrate the tooth structure and exert their effect by oxidizing the molecules that have caused discoloration in the tooth structure [9]. In addition, it has been shown that some adjunctive treatments might improve the efficacy of bleaching. According to Kurklu and Ozcan, the application of air-powder polishing before in-office bleaching treatment increased the efficacy of the bleaching procedure significantly [9]. Another study conducted by Camboni and Donnet concluded that air-powder polishing could clean tooth surfaces more deeply without causing damage to the enamel [14]. For the aforementioned reasons, we decided to use air-powder polishing before the bleaching procedure to improve the final aesthetic results. It is worth bearing in mind that air polishing should be cautiously used in practice since it generates aerosols of microorganisms and powder, and therefore personal protection including masks and eyewear should be worn by clinicians during the treatment.

It should be noted that reports regarding occupational tooth discoloration caused by metallic substances have decreased in recent years since the prevalence of these cases has declined due to the generally improved industrial hygiene in developed countries. However, these types of discoloration are still present in some circumstances due to the negligence of occupational safety protocols, including inadequate workplace ventilation and improper use of face masks.

## 4. Conclusion

Occupational exposure to bronze alloys may cause green staining on the tooth surface. A combination of rubber cap prophylaxis and air-powder polishing followed by in-office bleaching might be a conservative and effective treatment in cases of metallic tooth discoloration caused by copper.

## Data Availability

The data that support the findings of this study are available from the corresponding author upon reasonable request.
